# Amelogenesis Imperfecta and Early Restorative Crown Therapy: An Interview Study with Adolescents and Young Adults on Their Experiences

**DOI:** 10.1371/journal.pone.0156879

**Published:** 2016-06-30

**Authors:** Gunilla Pousette Lundgren, Anette Wickström, Tove Hasselblad, Göran Dahllöf

**Affiliations:** 1 Department of Dental Medicine, Division of Pediatric Dentistry, Karolinska Institutet, Stockholm, Sweden; 2 Center for Pediatric Oral Health Research, Stockholm, Sweden; 3 Department of Pediatric Dentistry, Public Dental Service, Dalarna County, Falun, Sweden; 4 Department of Thematic Studies, Child Studies, Linköping University, Linköping, Sweden; University of North Carolina at Chapel Hill, UNITED STATES

## Abstract

Patients with Amelogenesis imperfecta (AI) can present with rapid tooth loss or fractures of enamel as well as alterations in enamel thickness, color, and shape; factors that may compromise aesthetic appearance and masticatory function. The aim was to explore the experiences and perceptions of adolescents and young adults living with AI and receiving early prosthetic therapy. Seven patients with severe AI aged 16 to 23 years who underwent porcelain crown therapy participated in one-to-one individual interviews. The interviews followed a topic guide consisting of open-ended questions related to experiences of having AI. Transcripts from the interviews were analyzed using thematic analysis. The analysis process identified three main themes: *Disturbances in daily life*, *Managing disturbances*, and *Normalization of daily life*. These themes explain the experiences of patients living with enamel disturbances caused by AI and receiving early crown therapy. Experiences include severe pain and sensitivity problems, feelings of embarrassment, and dealing with dental staff that lack knowledge and understanding of their condition. The patients described ways to manage their disturbances and to reduce pain when eating or drinking, and strategies for meeting other people. After definitive treatment with porcelain crown therapy, they described feeling like a normal patient. In conclusion the results showed that adolescents and young adults describe a profound effect of AI on several aspects of their daily life.

## Introduction

**Fig 1:**
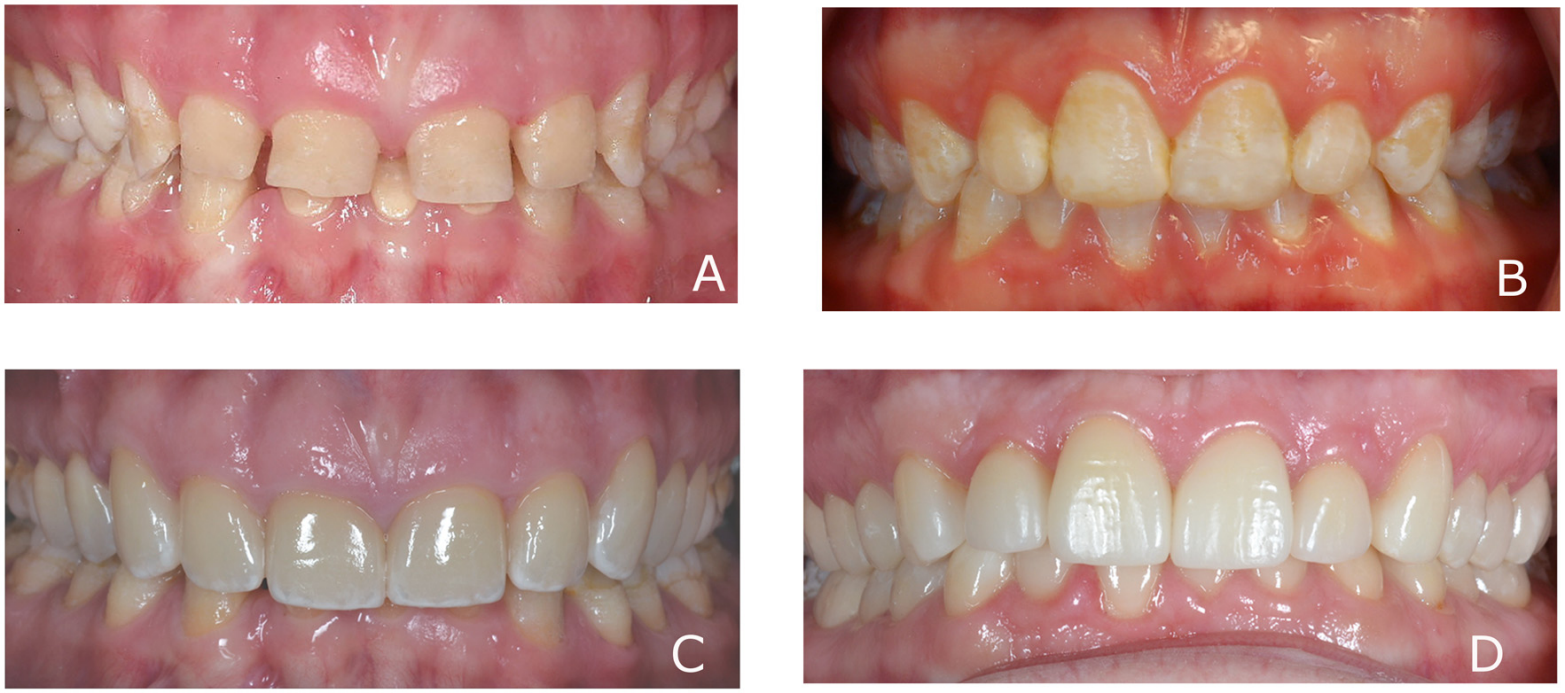
Clinical appearance before and after ceramic crown therapy. A) Hypoplastic type of AI before start of therapy, C) After crown therapy at 14 years of age, B) Hypomineralized form of AI before start of therapy, D). After crown therapy at 17 years of age.

Amelogenesis imperfecta (AI) is a rare, genetically determined defect in enamel mineralization. Prevalence of AI varies from 1:700 to 1:14,000 [1, 2]. The two major types of AI are hypoplastic and hypomineralized. Hypoplastic AI (Fig 1A) involves a quantitative reduction of enamel and presents with small teeth, a thin enamel layer, or pits or grooves in the teeth; hypomineralized AI (Fig 1B) is a qualitative disturbance in enamel formation with a normal quantity of enamel, resulting in a rough, discolored, often brown or yellowish tooth surface.

In the hypomaturated form of AI, often grouped together with the hypomineralized form, the enamel is of normal quantity but shows white spots [3]. The severity of enamel disturbances in patients with AI varies [4]. The most severely affected patients can present with rapid tooth loss or fractures of enamel as well as alterations in enamel thickness, color, and shape; all of these compromise aesthetic appearance and masticatory function. In combination with the tooth hypersensitivity commonly associated with AI, the extensive and lifelong restorative care that the condition entails is a burden to the patient [5]. Existing recommendations for AI treatment in patients under 19 years of age suggest considering direct restorations as temporary and planning for multiple replacements of restorations with permanent therapy postponed until adulthood [3, 6–8]. Patients with AI often experience negative aesthetic effects from tooth discoloration and reduced crown size and report significantly higher levels of social avoidance and distress than subjects without the condition [5]. The longevity of composite resin restorations in patients with AI is poor, necessitating many dental visits for replacements [9]. Patients with AI also experience poorer oral health related quality of life (OHRQoL) compared to normal controls [10] and AI also have impact on education, job satisfaction, and family building [5]. Early permanent therapy in young patients with AI has shown excellent clinical results [11]. We have also show an increased OHRQoL after crown therapy [10]. There is an obvious need for a deeper understanding of the impact of AI since questionnaires used to study OHRQoL do not reflect individual patients own experiences of living with AI its associated therapy burdens, and the outcome of early crown therapy. Our aim was to explore the experiences and perceptions of adolescents and young adults living with AI and receiving early prosthetic therapy.

## Materials and Methods

### Participants and methods

This study followed the Declaration of Helsinki guidelines and was approved by the Regional Ethics Review Board in Uppsala (Daybook number 2014/481).

Our study focused on the experiences of adolescents and young adults living with a severe form of AI and receiving early crown therapy. We asked seven patients with severe AI (five females and two males aged 16 to 23 years) who underwent porcelain crown therapy (with crown preparation beginning between 9 and 20 years of age) to participate in one-to-one individual interviews (Table 1).

**Table 1:** Clinical data of interviewed patients.

Name	Sex	Age at inter-view	Age at start of crown therapy	Number of crowns made	Preferred age for crown therapy	Type of AI	VAS score*
**Lisa**	F	16	13	10	Earlier	Hypoplastic	6.7/0.9
**Peter**#	M	21	9	20	As soon as possible	Hypomineralized	7.7/0.9
**Sara**	F	28	22	13	As soon as possible	Hypomaturation	6.3/0.6
**John**	M	20	17	16	17, it was right for me	Hypomineralized	1.0/0.0
**Eve**	F	19	14	6	12, it was two years late	Hypomaturation	1.1/0.0
**Livia**	F	23	17	24	As soon as possible	Hypoplastic	8.4/1.2
**Anna**	F	21	16	14	Earlier	Hypomineralized	7.0/1.3

^#^Peter: AI with frontal open bite

*before and after crown therapy

The subjects came from a convenience sample of patients near two major cities in Dalarna county. The participants received an informational letter telling them about the study and that they could withdraw from the interview at any time. All participants gave written consent to participate. For the one participant 16 years of age, verbal consent was obtained from parents over telephone, because the parents could not travel to the interview site. This verbal consent was documented in the records.

In-depth interviewing is a qualitative research technique that involves conducting intensive individual interviews with a small number of respondents to explore their perspectives on a particular item. In-depth interviews are useful for retrieving detailed information about a person’s thoughts and behavior or exploring new issues in depth [12]. The principal investigator (GPL) performed crown therapy for all patients while an independent psychologist (TH) who was experienced in cognitive behavioral therapy for dental phobia in children and adolescents conducted all interviews. The interviews were conducted away from the dental clinics and discussed the young patients’ experiences of living with AI and the dental treatment they received. The interviews followed a topic guide consisting of open-ended questions related to experiences of having AI. Three main themes were covered: as a child or adolescent, what is it like to have AI; what do you think about the dental care you have received? What is it like to live with AI after crown treatment? The interviewer (TH) let the patient take the lead and only asked follow-up questions when necessary or when the discussion lapsed. The interviews took between 27 and 63 minutes. An independent colleague who was experienced in qualitative research transcribed the interviews verbatim. All patients read and confirmed that the interviews were correctly reported in the transcripts. TH and GPL controlled the transcripts and compared them to the recorded data. AW and GD have previous experience of qualitative research [13, 14]. All patients appeared under pseudonyms in the text.

### Analysis of data

We analyzed the transcripts from the interviews using thematic analysis according to the method of Braun and Clarke [15]. Thematic analysis can be done using a “realist” approach, reporting the experiences and meanings that the subjects express, or a “constructionist” approach, reporting the different discourses the subjects use. This study followed a “contextualist” approach, in-between realism and constructionism, to interpret how participants make meaning and how the social context influences these meanings.

We followed the six steps proposed by Braun and Clarke [15]: First we familiarized ourselves with the data by listening to the recorded interview and reading and re-reading the transcribed interviews several times. We coded all interviews for interesting features, and made thematic maps of features related to the research questions. After sorting the data, we searched for potential themes and created a mind-map with possible themes for the data as a whole. For a theme to be valid, it had to satisfactorily answer the question “What is this expression an example of?” [15]. After identifying a set of candidate themes, we revised the themes and considered the validity of individual themes in relation to the data set. The analyzing process implied a movement back and forth between data and ideas of what would illuminate them. Two colleagues (GP, AW) together tested how the themes fitted with further data in a scrutinizing and iterative process. If no-consent was achieved around the themes and sub-themes the discussion involved GD and TH and consensus was achieved. Then we defined and re-defined the themes. Two colleagues (a psychologist and a pediatric dentist) reviewed the final analysis and confirmed the results. After this we were able to write the report. The transcribed interviews are in Swedish and can be made available to researchers on request to the corresponding author.

## Results

The analysis process elicited key concepts and identified three main themes: Disturbances in daily life, Managing disturbances, and Normalization of daily life. These themes explain the experiences of patients living with enamel disturbances caused by AI and receiving early crown therapy. In these main categories we identified several subthemes. Disturbances in daily life included the subthemes Dental pain and breakdown; Embarrassment and shame; and Lack of knowledge and understanding. Managing disturbances included Avoiding, hiding, and making excuses; Resigning or fighting; and Getting support. Finally, Normalization of daily life included Reduced pain and eating problems, Feeling assured and able to act “normally” in relationships, and Being a “normal” dental patient (Table 2).

**Table 2:** Themes and subthemes explaining the experiences of patients living with enamel disturbances caused by AI and receiving early crown therapy.

Themes	Subthemes
**Disturbance of daily life**	Dental pain and breakdown
	Embarrassment and shame
	Lack of knowledge and understanding
**Managing disturbances**	Avoiding, hiding and making excuses
	Resigning or fighting
	Getting support
**Normalization of daily life**	Reduced pain and eating problems
	Feeling assured and being able to act “normally” in relationships
	Being a “normal” dental patient

### Disturbances in daily life

This theme describes the insecurity of having teeth prone to disintegration, fractures, and increased sensitivity. Patients discussed their dental fear, not only about visiting the dentist but also of breaking or hurting their teeth. They also disclosed how embarrassing and ashamed they felt showing their teeth and how these feelings preoccupied their time and thoughts.

#### Dental pain and breakdown

Most patients reported severe sensitivity problems, especially sensitivity to warm and cool drinks, but also citrus fruits. Two patients mentioned increased sensitivity problems after eating citrus fruits that did not occur immediately after eating but the following day. Patients also mentioned delayed pain problems after tooth brushing when going out in cold conditions.

*I could not eat certain foods because I had a lot of pain in my teeth*. *It could appear totally unexpectedly and some days worse than others […] I couldn´t eat*, *for example*, *an orange*, *because it hurt and if I did anyway—the day after I couldn´t eat for a long time*, *and I couldn´t eat ordinary food either because it hurt*. *Lisa*, *16 years old*

Several patients mentioned rapid loss of enamel and fractures as great problems. They mentioned the fear of chipping the enamel or fear of pain as a part of daily life.

*They were so prone to damage*, *that*, *for example*, *sometimes when I bit into a fork*, *pieces of enamel would chip off*. *Anna*, *21 years old*

Another common problem was repeated loss of restorations after repair of fractured surfaces. This required additional dental treatment and its associated pain experiences.

*Then it was a lot of temporary restorations and such things—before I got my permanent crowns*, *temporary restorations breaking down again and so on … Peter*, *21 years old*

Patients experienced a variety of sensitivity problems, fractures, pain (immediate or delayed), and loss of restorations.

#### Embarrassment and shame

Patients with AI described several severe impacts on self-confidence. All patients expressed concerns about the appearance of their teeth and felt that they were different in a bad way. They mentioned comments from others about having disgusting teeth, dirty teeth, not having brushed their teeth, and not taking care of oral hygiene. Some patients remembered beginning to worry about their teeth at 5–6 years of age, but the most common age at which they reported the onset of problems with self-confidence was 10 years.

*I´ve been told about my bad teeth my whole life*, *they didn´t really know why—yes*, *I’ve heard I’ve got defective enamel*. *Lisa*, *16 years old*

Most of the patients felt ashamed and embarrassed about the appearance of their teeth and were afraid to show them to other people.

*If you have unhealthy teeth you feel unhealthy and well disgusting*. *Anna*, *21 years old*

One young patient had received her crowns at age 14 years. Without early crown therapy, she does not think she could have managed high school, because of the shame she felt previously about her teeth.

*It would have been different if I had had it done now*, *when I am 19*,. *I think it would have been a little different*, *because then I would have gone with it so long—all of high school and everything*, *[f*.*…*.!*] I would not have managed*, *looking like that*. *Eve*, *19 years old*

Feelings of shame and embarrassment are far-reaching according to the interviewees. These feelings preoccupied them with the condition of their teeth and created a sense of social insecurity. All patients were significantly preoccupied with the condition of their teeth.

All patients but one expressed a feeling that something was wrong and of being afraid of what others would think about them. They reported a social insecurity that grew with age.

*It was more and more difficult to find friends*, *as I got older*. *Livia*, *23 years old*

Patients reported that more and more of their daily and social life was occupied by thoughts of their dental problems.

*I always compared myself with others*, *or if I was talking with someone I thought of the appearance of their teeth […]*. *It was something occupying my thoughts*. *Anna*, *21 years old*

The patients described how their dental condition impacted their social relationships by making them embarrassed and promoting feelings of shame. In daily life they were often preoccupied with the condition of their teeth and the feelings it generated.

#### Lack of knowledge and understanding

Lack of patient knowledge is understandable as AI is a rare disease and its clinical manifestations vary. Not knowing about the disorder, feeling alone in having AI, and having nobody to inform you about the disease and treatment possibilities was a problem for patients without relatives with AI. Some patients mentioned a need for patient interest groups, especially those who did not having siblings or relatives with AI. Patients themselves felt it was difficult to fight for their own therapy. Five of seven patients had had problems explaining their condition to dental staff.

*I met dentists earlier—they only did things*, *without telling you*, *and if it hurts or you are bleeding or whatever—they didn’t say a word*. *John*, *20 years old*

It hurt patients to receive negative remarks about not brushing their teeth and accusations about eating a lot of sweets from dental staff with limited knowledge of AI. The patients had problems not being listened to by dentists. As one patient described:

*I was only a child … they thought they had the best knowledge*. *Livia*, *23 years old*

A verified diagnosis helped when explaining to general dentists about sensitivity, the need for anesthesia, and problems with dental restorations.

Sensitivity was more difficult to explain, and if the patient complained about increased sensitivity to dental staff. The staff had difficulty acknowledging the problem, as it was not visible (Fig 1B).

Several patients mentioned the need for more local anesthesia during dental treatment than what was offered. After dental visits, they did not feel as if their problems had been addressed or that their restorations would last. One patient developed a severe dental phobia after being treated without local anesthesia several times and not being listened to when crying because of the painful treatment.

*Before they realized they should use local anesthesia—it was unbearable*. *I remember one dentist he caused my dental phobia*. *Because he drilled and drilled and he drilled without local anesthesia*, *and it hurt so much*, *and he said a little more and a little more and—I was ready to hit him … Livia*, *23 years old*

Some patients mentioned problems with post-treatment sensations associated with various dental materials:

*It´s like a permanent tension in the tooth—the tooth gets more sensitive when restored with resin composite […] I have had huge problems finding a dentist who knows what they are dealing with—for example*, *they would like to restore with resin composite—I scream right out*, *they are not allowed to do that because it hurts*, *it really hurts in my teeth … glass ionomer was much better*, *then I can’t feel the restoration … and restorations fail very often in our teeth*. *Livia*, *23 years old*

The patient reported dentists not listening to her explanations, even if she said it would result in difficulties with pain afterwards, and she had to change restoration material only days after the first treatment. This was especially difficult because of the many different dentists in the clinic not communicating with each other or documenting the specific problems for this patient. It was also difficult for the patients to have their wishes respected if, for example, they asked the dentist not to blow air or use a dental suction device because of sensitivity problems.

The patients’ many and frequent dental visits caused problems at school. They had to try to explain their treatment needs to teachers and explain why they had to visit a specialist pediatric dentistry clinic and not a general dentist close by. They often felt distrusted.

*When I was younger it was really often*. *To tell them in school that you had to leave—they had to agree sort of*, *that you left*. *And sometimes it was once a month sort of … Peter*, *21 years old*

Patients with AI did not receive respect and trust. Furthermore, being questioned regarding their need for these frequent treatments seems to have added to their feelings of vulnerability.

### Managing disturbances

The patients developed various strategies for avoiding problems and embarrassments. All but one patient mentioned employing strategies to hide or cover their teeth or avoid specific foods when interacting with others. Some patients explained that they felt forced to accept the situation while others were fighting to be listened to. Having support from families, mothers, siblings, and even cousins was a strengthening factor.

#### Avoiding, hiding, and making excuses

Having to avoid specific foods due to chewing problems and pain was an issue that influenced the patients’ daily lives. Eating meals took longer because of sensitivity problems. Adolescents reported that they didn’t have enough time to finish their meals in time to join their friends, and sometimes went hungry.

*I had huge problems with my teeth*. *They were so sensitive both to warm and cold and if I bit a little wrong it hurt […] I couldn´t eat as fast as everybody else at school*, *it was as if I couldn´t chew as fast as others*. *I was always the last one to leave the canteen*. *My friends got tired—so I didn´t eat a lot either*. *Livia*, *23 years old*

The various strategies for dealing with daily life included covering the teeth with the lips, stretching the lips over the teeth to cover them, or even using a straw when drinking to avoid sensitivity problems. Drinking or eating without touching the teeth helped with sensitivity problems. Another strategy involved covering the mouth with something, such as a scarf, before going out in the cold air. One patient brushed her teeth early in the morning, rinsed with warm water, and then waited an hour before going out in the cold air to avoid sensitivity problems. To minimize the effects of aesthetic problems, patients tended to cover themselves, holding their hands over their mouths, using scarves, not showing their teeth, not laughing, and controlling their smiles.

*My teeth were different and I didn´t want to show them to avoid getting comments about them*, *especially when meeting new persons*. *I tried to avoid meeting new persons*, *but when I did meet new persons I tried to cover my teeth with my lips*, *tried not to laugh*, *and only smiled just a little*. *Anna*, *21 years old*

The esthetical problems patients faced included small teeth, fractured teeth, and yellowish or brown teeth, and all patients avoided being photographed when smiling or laughing. Strategies patients mentioned to avoid others’ comments included telling their best friends preemptively or avoiding having a lot of friends. One patient, a young adult, used to excuse herself and explain about her teeth before anyone else mentioned them in order to avoid comments.

#### Resigning or fighting

Some of the patients seemed to have grown resigned to others’ comments and to having to wait for treatment. Others got angry about the comments or seemed willing to fight to get early treatment. Dealing with the comments of others was often difficult and hurtful.

*“Friends and others have pointed out that my teeth are ugly—and I have heard a lot from others—that you don’t brush your teeth*, *that you do not care about oral hygiene … a lot of such comments and from new dental staff that I have to brush better—I tried to just leave it and let it be*. *Livia*, *23 years old*

A patient sometimes received comments when out dancing.

*Sometimes*, *when I was out dancing if someone made a comment on it I just went home*, *it spoiled the whole evening*, *I thought*: *“No*! *I am going home” … It was a really weak spot for me*. *When I think about it—I have no other complaints about my appearance*. *Sara*, *28 years old*

She also expressed anger.

*Who are they to judge me for having this very visible defect—what gives them the right to comment on that*? *Sara*, *28 years old*

Some of the patients seemed to accept their situation with resignation when informed that there was nothing to be done before adulthood.

*It was nothing to care about—it was only to accept*. *John*, *20 years old*

The stories of the interviewed patients showed that it seemed difficult for a young person to fight or demand treatment, especially when being informed that no permanent treatment is possible in combination with assurances that the dentist is providing the best treatment.

#### Getting support

The interviews identified parents, sometimes siblings, and even cousins with the same disorder as a supporting and strengthening factor for patients, particularly parents who would fight for their child’s treatment.

.. *but*, *it's just that my mother's been so*, *she's had to fight so much … and what I can remember*, *she's really had to talk [with the dentist] … when I would go to a new dentist*, *she would say*, *like*, *"no*, *stop*, *wait*, *first you need to know this"*, *and "you might have to do this in another way"*, *and … "think about what you are doing"*, *and … "the things you do are not always for yourself*, *[you can't] just think*, *'how bad can it be*?*' " Sara*, *28 years old*

The patients appreciated their parents speaking up for them during treatment. This could be, for example, demanding sedation and local anesthesia, or informing dentists about the condition. One patient who did not have AI in her family explained that lack of experience could be a problem, but also that she herself could be a resource for others.

*I haven’t met anyone that has the same problem—it might be easier to discuss*, *sort of—if you have something in common … If there is someone else wondering I could give them an answer*. *Lisa*, *16 years old*

According to the patients interviewed, it makes a difference to have support and information about the disease when discussing treatment possibilities, or in gaining hope for a solution and learning what kind of therapy others have gotten.

### Normalization of daily life

After crown therapy (Fig 1C and 1D), all patients reported significantly improved oral health. Not only had their experience of discomfort decreased, but also their esthetic problems. They felt they were treated in a more positive way and also felt and acted differently in a positive way themselves. They also reported that they were treated differently in dentistry after receiving crowns, more professionally and with respect. There was also an end to the problems of having to leave school or a job for dental appointments.

#### Reduced pain and eating problems

After treatment, many patients concluded there was a new possibility: “to feel without pain” in their teeth. Sensitivity to warm and cool drinks as well as to ice cream and citrus fruits decreased in all patients. Cold air was no longer unpleasant. Six patients reported being able to eat food that they could not eat before. The risk of enamel fractures was no longer a problem when chewing.

*The difference*? *Huge*! *Great—no sensitivity problems and I can sort of eat without being afraid of fractures*. *Anna*, *21 years old*

Loss of restorations and problems with many dental visits, often in combination with pain and sensitivity issues, were no longer problems. The patient with severe dental phobia reported:

*I learned to control my dental fear and to handle the dentists so that they listen to me*. *Livia*, *23 years old*

This patient reported that she learned strategies to control and cope with dental fear and also to communicate about her feelings with the dentist to create a good working relationship.

#### Feeling assured and being able to act “normally” in relationships

After crown therapy, patients mentioned comments from others (mostly family friends), including: that they seemed to be more satisfied with themselves; that they were more positive, smiling and laughing spontaneously; and that they smiled when taking photographs. Some of the patients felt so different that they imagined others appreciated them as a totally new person in a positive way. Patients mentioned that having AI and getting treatment strengthened them and no one felt ashamed or embarrassed after treatment.

*I feel like a normal person …*. *When I got the crowns*, *I forgot everything I hated before*. *Eve*, *19 years old*

Therapy solved esthetic problems, even when not all teeth received crown therapy. Several patients reported something similar to the following:

*I love my teeth—it went from being a huge problem to being nothing at all*. *Eve*, *19 years old*

Three patients mentioned it had changed their teeth from being a huge problem to nothing to think about at all. Others felt proud and felt they were privileged. A patient receiving crowns at 23 year of age said about receiving crowns that:

*This had affected me for so long that it continued to do so even after treatment*, *until I suddenly felt like everyone else—and could use it to my favor*. *Sara*, *28 years old*

All patients expressed greater self-confidence after crown therapy. Feeling more socially secure, relaxed, and not being occupied by thoughts of their teeth strengthened the patients. Relaxing, smiling, and daring to laugh were positive new experiences and interactions with friends became more normal and open.

*I can visit a café*, *laugh and smile with my friends and I don’t have to cover my mouth … Now I can concentrate on the issues when talking with another person—not only think of covering my teeth*. *Anna*, *21 years old*

The patient’s improved overall appearance and self-confidence was an asset at job interviews and their chances for getting a job or career was much greater. Some patient statements express the deep impact of crown therapy on having AI:

*How is it now*? *Well*, *my self-confidence—if I should have the teeth I had then it should be—tremendously … I should not have a girlfriend now*, *for example*. *It should be a problem when applying for jobs*. *The difference is tremendous—on every level*! *Peter*, *21 years old*

The patients reported feeling normal and focused on their interactions with others instead of thinking about their teeth and what others will think about them.

#### Being a “normal” dental patient

It was a new experience to feel like a “normal patient” when visiting the public dental service clinics. After crown therapy you are considered a normal patient and not a patient with AI and it was nice having fewer dental visits than before. Several patients expressed feelings similar to this:

*It feels like an end of all this running frequently to the dentist*. *John*, *20 years old*

One patient mentioned that, after crown therapy, it was rather fun coming to the public dental service and telling them how to treat her. But she had noticed that every time she had to “choose her words” carefully to get the dental staff to listen to her. Two patients said they had special knowledge after being treated at the pediatric specialist clinic and that they were proud to tell others about it and offered to support others with their treatment.

*Recently I finished the last check-ups*, *it has just been going on as long as I can remember—now it is a little strange not expecting to go there—it´s about a year to the next visit—it´s strange—but nice*. *Peter*, *21 years old*

However, being a “normal patient” in the context of having AI also includes worries that most patients probably do not have; worries about issues such as the need for replacing crowns later on.

*My mother frightened me—she lost her crowns a couple of times*, *I have had nightmares about losing my crowns*. *Anna*, *21 years old*

Patients also worried about their future offspring:

*You don´t know if this disease will affect your children*. *Eve*, *19 years old*

All patients were relieved when their dental visits and their role as patients were normalized. Nevertheless, their dental condition still created some worries about not being able to find a dentist with knowledge about their disease or the best treatment in the future.

## Discussion

The results of this study show the profound impacts on daily life experienced by children and adolescents affected with AI. The results give voice to these individuals and provide insight into what it means to live with AI when growing up. These results supplement our previous study showing a reduced OHRQoL in children and adolescents with AI [10]. Marshman et al. [16] pointed out that, while most studies use children as objects of research, few studies focus on children’s own views of their treatment. The patients included in this study had a long history of dental care and prior to treatment with ceramic crowns been treated with the older treatment paradigm using resin composite to cover surfaces affected by enamel hypomineralization or hypoplasia.

This study identified three major themes: disturbances in daily life, managing disturbances, and normalization of daily life. In the patients’ own voices, the themes describe the impact of AI on their daily life, how they cope with the consequences of the condition, and how early treatment with porcelain crowns changed their daily life.

Accumulating evidence is showing that patients with AI experience lower OHRQoL than patients without AI [5, 11, 17]. Bio-psychosocial factors explain the determinants of OHRQoL; these include symptoms, functional status, and general health perceptions within the context of individual and environmental characteristics [18].

Patients in this study reported heightened tooth sensitivity with painful experiences when eating or drinking warm or cold foods or drinks, or when going out into cold weather. They also reported pain reactions to restorative materials and more painful dental treatments without sufficient local anesthesia. The results are in line with a previous study in which AI patients scored 5.2 on a pain visual analogue scale [10] and a study by Coffield et al. [5] in which 82% of AI patients reported increased sensitivity compared with a control group. The results also showed that these painful stimuli can be unpredictable and that pain can occur a day after exposure.

All patients in this study expressed concerns about the appearance of their teeth and that they felt different in a negative way. They also said that the condition affected them in a negative way in daily life. They were preoccupied with thoughts about how the appearance of their teeth would affect people they met and what these people would think of them. Parekh et al. [19] reported that children and adolescents with AI exhibited concerns regarding aesthetics and function and expressed a high level of concern regarding the comments of other people and the self-consciousness associated with this. Contrary to the results in this study, Parekh et al. [20] reported that in focus group discussions, adolescents with AI did not appear to have any psychosocial effects of AI, which was opposite to their parent’s perceptions. This is probably an effect of the study setting; adolescents with AI do not want to discuss their difficulties in-group and with strangers.

Appearance becomes more important during adolescence. There are strong cultural pressures to conform to beauty ideals, and adolescents often become preoccupied with their own and others’ appearance [13, 21]. Seehra et al. [22] report bullying in association with certain malocclusions and this affects self-esteem and OHRQoL negatively. Children with visible differences have been found to be more likely to encounter discrimination and unsolicited negative attention from others [23]. Patients in our study also reported appearance-related teasing and bullying. This has been reported for several groups of children with appearances different from normal [24]. Fractures and loss of restorations resulted in many dental appointments that also affected school performance. Being questioned regarding the necessity for frequent dental appointments, often in front of others, was a burden for the patients.

AI is a rare disorder, and general dentists, hygienists, and dental assistants do not often meet patients with AI. Children and adolescents in this study describe a variety of problems with dental staff who either did not understand their condition and its consequences and blamed them for poor oral hygiene or did not understand their need for extra pain relief. Klingberg et al. [25] reported similar results in children with rare disorders, physical disabilities, and cognitive impairments where lack of knowledge, lack of understanding of patient needs, and lack of organizational support negatively affected quality of care. In order for dentists to provide successful care in situations that deviate from the norm requires them to handle professional uncertainty, to dare to face difficulties, and to work in a tolerant work environment.

Patients in this study also reported that dentists did not listen to them and their specific problems. Reports show that dental professionals try to normalize children with rare disorders in order to make their treatment situation more manageable [26]. This also applies to less apparent disabilities with which they are unfamiliar such as ADHD. For children and adolescents with AI to receive equal and fair treatment in dentistry, it is vital to consider the wishes of parents of children with disabilities, who identified five areas perceived as important qualities for dental teams: respect, involvement, continuity, knowledge, and availability [27].

Patients interviewed in this study reported several strategies to avoid the pain and increased sensitivity caused by AI, including hiding their condition from others and avoiding social situations where they risked unsolicited comments from others. Fear of negative judgments and social anxiety can result in social avoidance and interpersonal difficulties, which may impede the development of social skills and lead to isolation from peers [28]. Adult patients with AI reported higher scores on social avoidance and distress compared to those without the condition. They also reported higher fear of negative evaluation by others and lower self-esteem and mastery [5]. Some patients in this study mentioned avoiding social contacts in order not to have to explain their condition to others and risk negative remarks. A study of romantic relationships in young individuals with visible differences, such as facial skin conditions or cleft lip and palate, found that the individuals thought appearance was important, that they were worried that they were unattractive to others, had fears of negative evaluations and used strategies for concealment and avoidance [23]. This study also found factors that could provide a buffer against the impact of having a visible difference, factors such as having good social skills, realizing that the condition is a part of one’s identity, and valuing attributes other than physical appearance.

The daily experiences of AI patients reported in this study are the consequence of current treatment paradigms [6, 8]. Patients have been told that there is nothing to do except wait until adulthood and manage with temporary restorations in the meantime. Some patients responded to this with resignation; others, especially those who had parents, siblings, or cousins with the same condition, had the knowledge and power to fight for adequate treatment. This is a common situation for parents of children with disabilities, having to fight for their child’s right to treatment [28]. It is also evident that treatment and education from the specialist pediatric dentistry clinic empowered the patients with knowledge and a permanent definitive treatment.

We previously reported excellent results from a 2-year follow-up study of early rehabilitation with porcelain crowns in children and adolescents with AI [11]. We also reported a significant and clinically important increase in OHRQoL in children and adolescents after early porcelain crown therapy [10]. After crown therapy, pain and sensitivity problems decreased and it was possible to live without pain. Several patients said that, for the first time, they realized how much pain they had had and how it had affected their lives.

Pain experiences are associated with psychological distress. TMD [29] and self-reported dental pain [30] are conditions associated with depression. It is also evident that outpatient care addresses the psychosocial distress associated with visible differences poorly [31]. This experience of a decrease in pain complements the findings of a previous study in which we found a decrease in pain VAS score from 5.2 to 0.6 after porcelain crown therapy in AI patients [11]. After crown therapy, all patients reported that they were relieved and did not constantly think about how their teeth would appear to others. Patients received positive remarks from others, felt proud of their teeth, and became happier people, smiling and laughing more. Treatment also resulted in increased autonomy for the patients. Now they could eat and drink everything and at the same speed as their friends. Issues with their teeth changed from being a constantly present problem to no problem at all. Feeling like a normal, intact person strengthened the self-confidence of all patients.

Several patients reported that they had visited the dentist on several occasions previously, but their problems were never solved and they were repeatedly disappointed. These dentists, however, were only following existing guidelines. After the patients had received their porcelain crowns, they were regarded as normal dental patients, which was an entirely new but pleasant situation. Regarding the timing of crown therapy, most patients thought it should have been done at an earlier age. The patients suggested that if crown therapy began at 12 years of age, even if they were subjected to extensive prosthetic rehabilitation, it would all be worthwhile. The results underline the importance of listening and giving a voice to the child as an acting subject capable of speaking about his or her own health.

In this study we have used thematic analysis to study experiences and perceptions of children and adolescents with AI. We used a convenience sample of patients living close to two major cities in Dalarna. Strengths of the study include that all the patients we asked agreed to participate. Second, an experienced psychologist (TH) conducted the interviews and the transcripts show that it is the voices of the patients that are the material for analysis. We have also been able to triangulate the results with quantitative data from two other studies in this project regarding pain and sensitivity and the effect of crown therapy on OHRQoL. To ensure anonymity and confidentiality the interviews were conducted in a setting out of the dental clinic, patients living area is not disclosed and we used pseudonyms for the citations.

## Conclusion

The results of this study showed that adolescents and young adults described a profound effect of AI on several aspects of their daily life. Experiences included severe pain and sensitivity problems, feelings of embarrassment, and dealing with dental staff that lack knowledge and understanding of their condition. Furthermore, the patients described ways to manage their disturbances and to reduce pain when eating or drinking, and strategies for meeting other people. After definitive treatment with porcelain crown therapy, they described feeling like a normal patient. We also conclude that there is a strong recommendation for early crown treatment to manage pain and esthetic concerns.
